# Comparative study of ureteral access sheath versus suction access sheath in retrograde intrarenal surgery

**DOI:** 10.1186/s12894-025-01976-4

**Published:** 2025-12-19

**Authors:** Ting-Yi Chiang, Kau-Han Lee, Chia-Chih Hsieh, Zhi-Hao Chen, Steven K. Huang, Allen W. Chiu

**Affiliations:** 1 Division of Urology, Department of Surgery, Chi Mei Medical Center, No.901, Zhonghua Rd. Yongkang Dist., Tainan, 71004 Taiwan; 2 Department of Urology, Shin Kong Wu Ho-Su Memorial Hospital, Taipei, Taiwan

**Keywords:** Retrograde intrarenal surgery (RIRS), Suction ureteral access sheath (S-UAS), Traditional ureteral access sheath (UAS), Stone-Free rate (SFR)

## Abstract

**Introduction:**

Retrograde intrarenal surgery (RIRS) is a minimally invasive technique for managing renal and upper ureteral stones. Ureteral access sheaths (UAS) facilitate instrument access and reduce intrarenal pressure, but their impact on stone-free rates (SFR) remains debated. Suction ureteral access sheaths (S-UAS) offer improved flexibility and can be connected to suction devices, enhancing stone fragment removal. This study aims to compare the outcomes of traditional UAS and S-UAS in RIRS.

**Methods:**

A retrospective cohort study was conducted at Chimei Medical Center from January 2022 to December 2024, including 104 patients who underwent RIRS with either traditional UAS (*n* = 53) or S-UAS (*n* = 51). Baseline characteristics, operative time, stone-free rates (SFR), postoperative complications, and the need for auxiliary procedures were analyzed. Stone-free was defined as absence of residual fragments > 4 mm (and > 2 mm for stricter assessment) on KUB. Subgroup analyses assessed the impact of stone size, location, and number on outcomes.

**Results:**

Immediate clinically acceptable SFR was significantly higher in the S-UAS group (82.0%) compared to the traditional UAS group (60.4%) (*p* = 0.016). At one month, no significant difference was observed between the groups. The S-UAS group required fewer auxiliary procedures (5.9% vs. 22.6%, *p* = 0.015) and demonstrated a higher SFR for lower calyx stones (81.8% vs. 55.2%, *p* = 0.046). Operative time, hospital stay, and complication rates were similar between groups.

**Conclusion:**

The S-UAS facilitates efficient stone dust evacuation, improves immediate clinically acceptable SFRs, and reduces auxiliary procedure rates without increasing complications. These findings support its use in RIRS, particularly in select stone characteristics. Prospective studies with larger cohorts and standardized imaging follow-up are warranted to validate these results.

**Clinical trial number:**

not applicable.

**Supplementary Information:**

The online version contains supplementary material available at 10.1186/s12894-025-01976-4.

## Introduction

Retrograde intrarenal surgery (RIRS) has become a cornerstone in the management of renal and upper ureteral stones, offering a minimally invasive alternative to percutaneous nephrolithotomy (PCNL). According to the European Association of Urology (EAU) guidelines, RIRS is recommended for kidney stones < 20 mm in size and, in certain cases, for lower pole stones >10 mm [1]. A critical component of successful RIRS is the use of ureteral access sheaths (UAS), which facilitate repeated instrument access, reduce intrarenal pressure, and enhance irrigation flow during surgery [2]. However, studies have shown that the use of a UAS does not significantly improve stone-free rates (SFR) or operative duration [2]. The limitations of traditional UAS, particularly in effectively clearing stone fragments, have driven the development of innovative solutions, such as the suction ureteral access sheath (S-UAS).

The S-UAS is distinguished by its excellent flexibility and deformability at the tip, allowing it to passively bend in alignment with the flexible ureteroscope. Furthermore, the S-UAS can be connected to a vacuum suction device, enabling efficient removal of residual stone fragments while further reducing intrarenal pressure. This design has the potential to improve SFR and minimize complications associated with retrograde intrarenal surgery. Studies have demonstrated the superior performance of S-UAS compared to traditional UAS, with Zhu et al. reporting significantly higher SFRs (81.3% versus 49.4%) [3], and Wang et al. showing similar improvements (88.73% versus 75.68%), along with a reduction in postoperative fever rates [4].

Despite its theoretical advantages, the benefits of the S-UAS in addressing variations in stone size, location, and complexity have been rarely explored. This study aims to share our experience in managing upper tract stones by retrospectively comparing the outcomes of traditional UAS and S-UAS in RIRS. Key outcomes assessed include immediate and delayed SFRs, operative time, postoperative complications, and the necessity for auxiliary procedures. By performing detailed subgroup analyses, this study seeks to provide a comprehensive evaluation of the clinical utility of S-UAS.

## Methods

This retrospective cohort study was conducted at Chimei Medical Center between January 2022 and December 2024. It aimed to compare the efficacy and safety of traditional UAS and S-UAS in RIRS. Patients were identified from electronic medical records and included if they met the following criteria: adults aged 18 years or older with renal or upper ureteral stones, confirmed by non-contrast computed tomography (CT) imaging, with stone diameters ≤ 30 mm. Patients with abnormal urinary tract anatomy (e.g., horseshoe kidney), active urinary tract infections, contraindications for RIRS, or those undergoing concurrent surgical procedures such as PCNL were excluded. Additionally, cases with incomplete data or failure to complete the assigned procedure were excluded.

A total of 121 patients were initially identified, but after exclusions for reasons such as anatomical challenges or procedural changes, 104 patients were included in the final analysis. These were divided into the traditional UAS group (*n* = 53) and the S-UAS group (*n* = 51). Group assignment was determined by the time period and availability of the S-UAS device. The S-UAS became available at our center in April 2024; hence, patients treated before April 2024 received the standard UAS, while those treated from April 2024 onward generally received the S-UAS. This sequential introduction means that allocation was not randomized, and surgeon preference or case selection may have influenced UAS type in some overlapping months. We acknowledge this as a potential source of selection bias. Baseline demographic and clinical data, including age, gender, body mass index (BMI), stone characteristics (location, size, and density), and preoperative laboratory values, were collected.

All surgeries were performed by experienced urologists using standardized protocols under general anesthesia. Preoperative imaging consisted of 2-mm non-contrast CT scans to assess stone size and density. Stone size was defined by both the maximum diameter of the largest stone and the cumulative sum of all stone diameters. Stone density was measured in Hounsfield units (HU). Residual fragment size was estimated on KUB by measuring the longest axis of visible opacity, as evaluated by both a radiologist and a urologist. Traditional UAS cases predominantly employed manual irrigation with a 50 cc syringe and nitinol stone baskets for fragment retrieval. In contrast, the S-UAS group used a suction-assisted sheath with adjustable suction pressures (50–100 mmHg) to facilitate stone aspiration during fragmentation.

A flexible ureteroscope was used in all cases (7.5 Fr Pusen™, 8.6 Fr WiScope™, or 9.5 Fr LithoVue™). Stone fragmentation was performed using a Holmium: YAG laser (Quanta Litho 60 W, 200 μm fiber), with settings adjusted based on stone characteristics to optimize dusting. Typical dusting parameters included 0.8 J at 30 Hz with long pulse duration, while higher energy settings (0.8–1.0 J, 30–40 Hz) were used for harder or larger stones. Popcorning (0.8 J, 40 Hz) was employed to reduce residual fragments when appropriate. In the traditional UAS group, gravity irrigation (height-adjusted) was used with intermittent manual flushing to improve visibility. In contrast, the S-UAS group underwent lithotripsy under continuous suction (− 120 mmHg) integrated into the access sheath, enabling active evacuation of dust and fine fragments. When the operative field was obscured (e.g., snow-globe effect), suction was transiently increased to restore clarity, with irrigation pressure maintained between 200 and 300 mmHg.

Most procedures used a 7.5 Fr ureteroscope with a 10/12 Fr sheath, though sheath size was adjusted according to ureteral anatomy and stone characteristics. In the suction UAS group, 7.5, 8.5, and 9.2 Fr scopes were used in 51.0%, 19.6%, and 29.4% of cases, paired with 10/12 Fr (30, 58.8%), 11/13 Fr (17, 33.3%), and 12/14 Fr (3, 5.9%) sheaths, respectively. In the traditional UAS group, a 9.5 Fr disposable scope was used with an 11/13 Fr sheath. This description addresses potential bias from instrument heterogeneity.

In traditional UAS cases, surgeons primarily used dusting to fragment stones into passable particles, with baskets employed as needed for larger or inaccessible fragments. In the S-UAS group, continuous suction facilitated effective dust removal, reducing reliance on baskets. Larger fragments were either further fragmented or retrieved by withdrawing the scope under suction. Baskets were occasionally used, particularly for calyceal stones beyond the reach of the flexible S-UAS. Although basket use appeared qualitatively lower in the S-UAS group, exact usage was not prospectively recorded, limiting quantitative comparison.

At the conclusion of each procedure, endoscopic inspection was performed to assess stone clearance. A 6 Fr double-J ureteral stent was routinely placed to maintain drainage and facilitate the passage of residual fragments. Postoperative evaluation included kidney-ureter-bladder (KUB) radiography within 24 h to assess residual stones and confirm stent positioning. Stents were typically removed at one month postoperatively in an outpatient setting. Timing of stent removal was consistent across both groups, unless altered by specific clinical indications, ensuring that differences in stone clearance were not attributable to variations in stent dwell time.

Primary outcomes included the immediate and one-month SFRs. While there is no universally accepted definition of “stone-free,” prior studies variably define it as complete absence of fragments on CT or as clinically insignificant residual fragments (CIRF) ≤ 4 mm or ≤ 2 mm on KUB [5, 6]. In our study, immediate SFR was defined as the absence of residual fragments >4 mm on KUB, with a stricter analysis also performed using a >2 mm threshold. The zero-fragment definition was not applied due to the limited sensitivity of our imaging modality. Follow-up imaging at approximately one month postoperatively was performed using KUB to assess stone clearance after potential spontaneous passage. Non-contrast CT was not routinely used at follow-up due to cost and radiation exposure concerns, limiting the detection of submillimeter residuals. Low-dose NCCT was used in 12% of cases with equivocal KUB results.

Secondary outcomes included operative time (defined from ureteroscope insertion to stent placement), hospital stay duration, auxiliary procedures, and complications. Auxiliary procedures were defined as any unplanned secondary ureteroscopy for residual/obstructive fragments (URSSM) or extracorporeal shock-wave lithotripsy (ESWL) performed after the index RIRS. Timing was left to the treating surgeon’s discretion and therefore was not protocol-restricted to the 1-month imaging visit. Statistical analyses were performed using SPSS version 25.0. Continuous variables were compared using independent t-tests or Mann-Whitney U-tests as appropriate, while categorical variables were analyzed using Chi-square or Fisher’s exact tests. Subgroup analyses explored outcomes based on stone size, location, and type.

## Results

Baseline demographic and clinical characteristics, including age, gender distribution, BMI, comorbidities, American Society of Anesthesiologists (ASA) classification, stone laterality, stone type, stone location, and stone size, were comparable between the two groups, with no statistically significant differences observed. The mean stone diameter was 14.27 ± 6.96 mm in the traditional UAS group and 13.74 ± 6.53 mm in the S-UAS group (*p* = 0.689). The preoperative serum creatinine levels and initial positive urine culture rates were also similar between groups. (Table 1)

**Table 1:** Baseline characteristics of participants.This table includes age, sex distribution, body mass index (BMI), comorbidities, ASA classification, laterality, stone type and location, stone size and density, preoperative laboratory values (serum creatinine and WBC), hydronephrosis grade, and pre-stenting status. Statistical comparisons were made between the traditional UAS and S-UAS groups

	Traditional UAS (*n* = 53)	S-UAS (*n* = 51)	*p* value
Age (yr)	57.30 ± 13.82	61 ± 12.21	0.151
Gender, *n* (%)			0.168
Male	38 (71.7%)	30 (58.8%)	
Female	15 (28.3%)	21 (41.2%)	
BMI (kg/m2)	26.74 ± 5.25	26.29 ± 3.98	0.625
Comorbidities, *n* (%)			0.625
pertension	3 (5.7%)	2 (3.9%)	
Diabetes	13 (24.5%)	14 (27.5%)	
Hypertension and diabetes	9 (17.0%)	4 (7.8%)	
Multiple	12 (22.6%)	11 (21.6%)	
ASA classification, *n* (%)			0.429
I	1 (1.9%)	2 (3.9%)	
II	15 (28.3%)	20 (39.2%)	
III	36 (67.9%)	29 (56.9%)	
IV	1 (1.9%)	0	
Laterality, *n* (%)			0.133
Right	18 (34.0%)	10 (19.6%)	
Left	20 (37.7%)	18 (35.3%)	
Bilateral	15 (28.3%)	23 (45.1%)	
Stone type, *n* (%)			0.331
Single	15 (28.3%)	19 (37.3%)	
Multiple	38 (71.7%)	32 (62.7%)	
Stone location, *n* (%)			0.376
Renal pelvis	3 (5.7%)	3 (5.9%)	
Upper pole	9 (17.0%)	6 (11.8%)	
Lower pole	29 (54.7%)	22 (43.1%)	
Proximal ureter	3 (5.7%)	8 (15.7%)	
Multiple or staghorn	9 (17.0%)	12 (23.5%)	
Largest stone diameter (mm)	14.27 ± 6.96	13.74 ± 6.53	0.689
Sum of stones diameter (mm)	25.56 ± 16.01	23.39 ± 15.28	0.482
CT value of stone (HU)	792.7 ± 255.5	753.4 ± 379.7	0.541
Pre-op serum Cr level (µmol/l)	1.08 ± 0.36	1.24 ± 0.67	0.122
Pre-op serum WBC count (10^3^/µl)	9.76 ± 3.58	8.09 ± 3.22	0.014
Initial positive urine culture, n (%)	10 (18.9%)	16 (31.4%)	0.141
Pre-stenting, *n* (%)			0.325
Double J	4 (7.5%)	5 (9.8%)	
PCN	3 (5.7%)	7 (13.7%)	
Grade of hydronephrosis, *n* (%)			0.901
G0	11 (21.2%)	11 (22.0%)	
G1	20 (38.5%)	21 (42.0%)	
G2	11 (21.2%)	12 (24.0%)	
G3	8 (15.4%)	5 (10.0%)	
G4	2 (3.8%)	1 (2.0%)	

### Primary outcomes

The immediate clinically acceptable stone-free rate (SFR), defined as the absence of residual stones > 4 mm on postoperative imaging, was significantly higher in the S-UAS group (82.0%) compared to the traditional UAS group (60.4%) (*p* = 0.016). When considering a stricter threshold of 2 mm for clinically acceptable SFR assessment, the S-UAS group still demonstrated superior clearance (70.0% vs. 50.9%, *p* = 0.048). At one month postoperatively, clinically acceptable SFR at the 4 mm threshold remained higher in the S-UAS group (85.7% vs. 75.5%), but this difference did not reach statistical significance (*p* = 0.193). Similarly, the one-month clinically acceptable SFR at the 2 mm threshold was comparable between groups (73.5% vs. 67.9%, *p* = 0.539). (Table 2)

**Table 2:** Primary and secondary outcomes.Outcomes assessed include immediate and 1-month SFR with 4 mm and 2 mm thresholds, operative time, postoperative hospital stay, need for auxiliary procedures, complication types and rates, postoperative renal function and WBC count, and stone composition distribution. Statistical comparisons are reported between the two groups

	Traditional UAS (*n* = 53)	S-UAS (*n* = 51)	*p* value
SFR (4 mm), n (%)	32 (60.4%)	41 (82.0%)	0.016
1 month SFR (4 mm), *n* (%)	40 (75.5%)	42 (85.7%)	0.193
SFR (2 mm), n (%)	27 (50.9%)	35 (70.0%)	0.048
1 month SFR (2 mm), *n* (%)	36 (67.9%)	36 (73.5%)	0.539
Operative time (min)	98.4 ± 40.85	95.9 ± 49.8	0.781
Post-op hospital stays (days)	2.79 ± 2.32	3 ± 3.69	0.733
Auxiliary procedure, *n* (%)	12 (22.6%)	3 (5.9%)	0.015
Complications, *n* (%)			0.383
Urosepsis	2 (3.8%)	1 (2.0%)	
Pneumonia	0	2 (3.9%)	
mucosal tear	2 (3.8%)	0	
Bleeding	1 (1.9%)	0	
ureteral stricture	0	1 (2.0%)	
Stone composition, *n* (%)			0.232
Calcium oxalate	25 (50.0%)	28 (54.9%)	
Calcium phosphate	2 (4.0%)	7 (13.7%)	
Calcium oxalate + Calcium phosphate	17 (34.0%)	9 (17.6%)	
Uric acid	4 (8.0%)	5 (9.8%)	
Calcium oxalate + Uric acid	2 (4.0%)	1 (2.0%)	
Struvite stone	0	1 (2.0%)	
Post op serum Cr level (µmol/l)			
Day 1	1.03 ± 0.36	1.05 ± 0.46	0.837
3 months	1.12 ± 0.48	1.21 ± 0.76	0.679
Post op serum WBC count (10^3^/µl)	11 ± 3.18	11.06 ± 4.56	0.94

### Secondary outcomes

Operative time did not differ significantly between the two groups, with a mean duration of 95.9 ± 49.8 min in the S-UAS group and 98.4 ± 40.85 min in the traditional UAS group (*p* = 0.781). Postoperative hospital stay was also comparable, with a mean duration of 3.0 ± 3.69 days in the S-UAS group and 2.79 ± 2.32 days in the traditional UAS group (*p* = 0.733). Notably, the need for auxiliary procedures, such as extracorporeal shockwave lithotripsy (ESWL) or secondary endoscopic procedures, was significantly lower in the S-UAS group (5.9%) compared to the traditional UAS group (22.6%, *p* = 0.015).

Regarding safety outcomes, the overall complication rates were similar between groups, with no statistically significant differences. Urosepsis was observed in 2.0% of patients in the S-UAS group and 3.8% in the traditional UAS group. Mucosal tear occurred in 3.8% of patients in the traditional UAS group, whereas no cases were reported in the S-UAS group. Bleeding complications were minimal, with a single case (1.9%) reported in the traditional UAS group and none in the S-UAS group. One case of ureteral stricture was noted in the S-UAS group.

Postoperative renal function was assessed through serum creatinine measurements on postoperative day 1 and at three months. No significant differences were observed between the groups, suggesting that the use of S-UAS did not negatively impact renal function recovery. (Table 2)

### Subgroup analysis

A subgroup analysis was performed to evaluate the efficacy of S-UAS in different stone sizes, stone locations, and stone burden.

#### Stone size (Cutoff at 15 mm)

For patients with large stones (> 15 mm), the immediate SFR was significantly higher in the S-UAS group (81.3%) compared to the traditional UAS group (40.0%) (*p* = 0.018). This trend was not observed in patients with stones ≤ 15 mm, where SFR differences between the two groups were not statistically significant. (Supplement Table S1)

#### Stone size (Cutoff at 20 mm)

Patients were stratified based on sum of stone size, with a cutoff value of 20 mm to assess whether S-UAS is particularly beneficial for larger stone burdens. In patients with stones > 20 mm, the immediate SFR was significantly higher in the S-UAS group compared to the traditional UAS group. In contrast, for patients with stones ≤ 20 mm, SFR was not significantly different between the two groups. (Supplement Table S2)

#### Stone number

When analyzing the impact of stone number, the S-UAS group showed significantly better outcomes in patients with single stones, achieving a 100% immediate SFR compared to 66.7% in the traditional UAS group (*p* = 0.008). However, in cases of multiple stones, SFR was higher in the S-UAS group (71.9%) compared to the traditional UAS group (57.9%), but the difference was not statistically significant (*p* = 0.224). (Supplement Table S3)

#### Stone location

The S-UAS group demonstrated a significant advantage in clearing lower calyx stones, with an immediate SFR of 81.8% compared to 55.2% in the traditional UAS group (*p* = 0.046). For stones located in other areas, including the renal pelvis, upper/middle calyx, upper ureter, or staghorn/multiple locations, no significant differences in SFR were found between the groups. (Supplement Table S4)

#### Postoperative white blood cell (WBC) counts

To further explore the postoperative inflammatory response, we compared preoperative and postoperative WBC counts. While preoperative WBC levels were slightly lower in the S-UAS group, postoperative WBC changes were not significantly different between groups, suggesting that S-UAS did not result in a markedly different inflammatory or infectious response compared to traditional UAS.

## Discussion

This study highlights the real-world clinical relevance of the S-UAS in enhancing immediate stone clearance during RIRS compared to traditional UAS. Our retrospective analysis of 104 patients demonstrated a significantly higher immediate stone-free rate in the S-UAS group while maintaining comparable complication rates. Additionally, the need for auxiliary procedures was significantly lower in the S-UAS group, indicating improved procedural efficiency. Notably, our subgroup analysis revealed that S-UAS was particularly effective in managing large stones, single stones, and lower calyx stones, providing valuable guidance for optimizing clinical decision-making and surgical approaches.

The implementation of S-UAS in RIRS has demonstrated increased efficacy, primarily through mechanisms such as the removal of debris, which enhances intraoperative visibility by mitigating the “snow-globe effect” and facilitating the identification of concealed fragments. This improvement in visualization contributes to higher immediate clinically acceptable SFRs [4]. Suction UAS facilitates the removal of fine fragments during lithotripsy, potentially reducing reliance on basket retrieval and minimizing residual dust that may contribute to stone recurrence or postoperative symptoms. A recent systematic review by Moretto et al. noted the lack of a standard definition for “stone dust,” though it is generally considered to be sub-millimeter or aspiratable particles [7]. By incorporating suction, the S-UAS addresses this critical but often overlooked aspect of dust management in RIRS. Fragment size significantly influences suction efficiency. Our findings suggest that S-UAS is most effective when stones are thoroughly dusted, particularly in single-stone cases. In contrast, multiple or partially fragmented stones may leave larger remnants less amenable to suction. Chan et al. demonstrated that particles ≤ 0.25 mm are optimal for consistent evacuation using a 5.1 Fr suction-enabled ureteroscope. Accordingly, we prioritize dusting settings to maximize synergy with the suction function of S-UAS [8]. 

Recent benchtop studies have evaluated the technical performance of S-UAS systems. Madden et al. demonstrated that a scope-to-sheath diameter ratio of 0.53–0.63 enabled successful aspiration of fragments up to 2 mm, with optimal suction for fragments < 0.5 mm during continuous lithotripsy, and “pull-and-suck” techniques proving effective for 1–2 mm fragments [9]. In our cohort, the most common configuration was a 10/12 Fr sheath (58.8%) with a 7.5 Fr ureteroscope (29.4%), yielding a ratio of ~ 0.75. These findings underscore the importance of matching fragmentation technique and sheath configuration to maximize suction efficacy and stone clearance.

Previous studies have reported increased SFRs both immediately and at three months postoperatively, typically defining clinically acceptable SFR as residual fragments smaller than 4 mm on KUB imaging, with the overall SFR ranged higher from 87.5% upwards [10–13]. In our study, we adopted a more stringent criterion by defining clinically acceptable SFR as the absence of residual fragments larger than 2 mm on KUB imaging. This approach revealed a significant improvement in immediate SFR with the use of S-UAS. However, this significance did not persist at the one-month follow-up. This observation may be attributed to the natural clearance of small residual fragments through urination in patients treated with traditional UAS, thereby diminishing the immediate advantage observed with S-UAS. Additionally, the non-standardized timing of auxiliary procedures limits the interpretability of delayed SFR as a pure outcome measure.

The aspiration capability of S-UAS not only enhances immediate stone clearance but also reduces the incidence of steinstrasse and the necessity for auxiliary procedures such as ESWL or secondary ureteroscopy. This reduction in additional interventions potentially contributes to a smoother postoperative recovery and an improvement in patients’ quality of life [3, 4]. Moreover, the effective aspiration of debris by S-UAS minimizes the need for fragment extraction using stone baskets, thereby streamlining the procedure. However, basket use was not prospectively recorded in this study. Thus, while the qualitative impression was of lower basket dependence in the S-UAS group, the absence of quantitative data limits definitive conclusions.

While other studies have reported a decrease in operative time with the use of S-UAS [13, 14], our findings did not demonstrate a significant reduction in surgery duration. This discrepancy may result from the additional time required to maneuver the suctional tip into the lower calyx. With increased experience and proficiency at our center, it is anticipated that this aspect of the procedure may become more efficient, potentially leading to shorter operative times in future studies.

Advancements in RIRS technology have led to an increasing preference for endoscopic management of larger renal calculi. A feasibility study evaluating a novel tip-flexible suction ureteral access sheath (NTFS-UAS) in combination with flexible ureteroscopic lithotripsy for kidney stones ≥ 30 mm demonstrated promising outcomes, reporting immediate and 1-month SFRs of 83.98% and 85.44%, respectively. Multivariate analysis identified stone size (≥ 50 mm), multiple stones, and severe hydronephrosis as independent risk factors negatively affecting stone clearance rates [15]. 

Our subgroup analysis further supports these findings, indicating that patients with a single stone larger than 15 mm or a cumulative stone diameter exceeding 20 mm achieved significantly better clinically acceptable SFRs with the use of S-UAS. Moreover, improved SFR was observed for single stones, consistent with previous research. This suggests that S-UAS may be particularly beneficial in patients with a substantial stone burden, thereby expanding the applicability of RIRS for managing larger renal stones.

Furthermore, Chen et al. compared suctioning semirigid ureteroscopic lithotomy (Sotn-URSL) with minimally invasive percutaneous nephrolithotomy (mPCNL) and reported an SFR of 84.6% in the Sotn-URSL group versus 92.3% in the mPCNL group, with the difference not reaching statistical significance (*P* = 0.34). Additionally, the Sotn-URSL group experienced shorter hospital stays and fewer complications compared to the mPCNL group [16]. Based on these findings, S-UAS could be considered a viable alternative to mPCNL for patients with single renal stones measuring 20–50 mm, offering a less invasive option with comparable stone clearance outcomes.

RIRS presents significant challenges in the management of lower calyceal stones due to the acute infundibulopelvic angle, which limits access and increases the need for frequent stone basket manipulation. These factors can complicate stone retrieval and prolong operative time, potentially impacting surgical efficiency and outcomes [17, 18]. In our study, the use of S-UAS resulted in a significantly higher clinically acceptable SFR for lower calyx stones compared to traditional UAS. The incorporation of a bendable tip in S-UAS facilitates precise navigation into the targeted calyx, overcoming anatomical challenges associated with the lower pole. Additionally, the continuous suction mechanism enhances fragment evacuation, reducing residual stone burden and improving immediate clearance. Notably, prior studies evaluating tip-bendable UAS without suction have reported lower SFRs and increased reliance on stone baskets in the lower calyx subgroup [19]. This suggests that while tip flexibility enhances maneuverability, the suction mechanism provides an additional advantage in fragment clearance.

The application of S-UAS during RIRS offers significant advantages in managing intrarenal pressure (IRP) and enhancing irrigation flow. Effective regulation of IRP is crucial, as elevated pressures can lead to pyelovenous backflow, increasing the risk of mucosal injury within the collecting system and facilitating the reabsorption of irrigation fluid containing bacteria and endotoxins, potentially resulting in postoperative infectious complications such as sepsis [20, 21]. In our study, the use of S-UAS was associated with comparable overall complication rates to traditional UAS, with a lower incidence of postoperative urosepsis observed in the S-UAS group. Specifically, one patient in the S-UAS group developed urinary retention and fever following Foley catheter removal, which was later diagnosed as underactive bladder. In contrast, two patients in the traditional UAS group developed postoperative urinary tract infections.

Notably, our study found no significant elevation in postoperative WBC counts in the S-UAS group compared to the traditional UAS group, indicating that the rate of sepsis was controlled and aligned with previous studies. This aligns with findings from other studies, such as one that demonstrated the application of suctioning UAS during flexible ureteroscopy was associated with lower incidence of postoperative fever or systemic inflammatory response syndrome (SIRS) [22]. These observations underscore the importance of patient selection and preoperative optimization to mitigate the risk of infectious complications. Further studies are warranted to monitor IRP during S-UAS use and to establish protocols that ensure patient safety and optimal outcomes.

Stone composition can modulate laser-dusting efficiency and, by extension, the performance of suction evacuation. Hard calculi such as calcium-oxalate monohydrate or cystine fracture into coarser fragments that are less readily aspirated than softer stones like uric acid or struvite [23]. In our cohort, laboratory analysis showed comparable distributions of stones between the S-UAS and conventional UAS groups; subgroup evaluation revealed no statistically significant clinically acceptable SFR differences attributable to composition. These findings indicate that the clinical advantage of S-UAS over standard sheaths is preserved across common stone types, with no evidence that particular compositions diminish its efficacy.

Several limitations should be acknowledged. First, the retrospective, non-randomized design introduces potential selection bias. Group allocation was primarily time-based (pre- vs. post-introduction of S-UAS), which may coincide with other temporal variables such as increasing surgeon experience or technological refinement. Second, the overall sample size—particularly in subgroup analyses—was limited, increasing the risk of type II error and rendering some findings exploratory. Although no a priori power calculation was performed due to the retrospective nature of the study, we conducted a post hoc analysis based on our primary outcome (immediate clinically acceptable SFR), which yielded an estimated power of approximately 68.7%. While below the conventional 80% threshold, the observed effect size suggests a clinically meaningful difference that warrants confirmation in larger prospective trials. Third, our definition of clinically acceptable SFR relied on KUB imaging, which may underestimate residual fragments < 2 mm. Non-contrast CT was reserved for equivocal cases due to radiation exposure and cost, in accordance with Taiwan’s national health insurance policy. However, this limitation applied equally to both groups, preserving the validity of comparative outcomes. Fourth, intrarenal pressure and fluid absorption were not measured; including these parameters would strengthen conclusions regarding the safety profile of S-UAS. Fifth, operative technique—such as laser settings and basket use—was not standardized and varied by surgeon, potentially limiting reproducibility. Lastly, the absence of long-term follow-up (> 3 months) precludes assessment of stone recurrence or delayed complications.

Future research should employ a prospective, randomized controlled design with a larger cohort to validate these findings and reduce biases. Additionally, studies focusing on intrarenal pressure monitoring during RIRS with S-UAS could provide valuable insights into its safety profile and ability to prevent adverse events such as pyelovenous backflow and sepsis.

## Conclusion

The integration of S-UAS into RIRS significantly improved immediate clinically acceptable stone-free rates and reduced the need for auxiliary procedures compared to traditional UAS, without increasing complication rates. These benefits were most pronounced in patients with large, single, or lower calyx stones. By enhancing fragment evacuation and visibility during lithotripsy, S-UAS demonstrated clinical utility across a range of stone burdens and compositions. Although no significant difference in operative time was observed, the effective aspiration of stone dust suggests procedural efficiencies that may become more evident with greater experience. The findings support the role of S-UAS as a valuable adjunct in RIRS, particularly in complex or high-volume stone cases. Prospective, randomized studies are warranted to confirm these results and further define optimal indications for S-UAS use.

## Supplementary Information

Supplementary Material 1

## Data Availability

The datasets generated and analyzed during the current study are available from the corresponding author on reasonable request.

## Abbreviations

RIRS: Retrograde Intrarenal Surgery
UAS: Ureteral Access Sheath
S-UAS: Suction Ureteral Access Sheath
SFR: Stone-Free Rate
PCNL: Percutaneous Nephrolithotomy
CT: Computed Tomography
HU: Hounsfield Unit
WBC: White Blood Cell
IRP: Intrarenal Pressure
ASA: American Society of Anesthesiologists
KUB: Kidney, Ureter, and Bladder radiograph
URSSM: Ureterorenoscopic Stone Surgery Manipulation
