# Supplementary material for: Comparative study of ureteral access sheath versus suction access sheath in retrograde intrarenal surgery
Source: BMC Urol. 2025 Dec 19;25:303. doi: 10.1186/s12894-025-01976-4 (PMC12717737; doi:10.1186/s12894-025-01976-4)
Supplement: Supplementary file 1 — Supplementary Material 1 [file 12894_2025_1976_MOESM1_ESM.docx]

**Supplement Table S1** Subgroup analysis of outcomes based on maximal stone size with cutoff value of 15mm

|  | Largest stone > 15mm | | | Largest stone <15mm | | |
| --- | --- | --- | --- | --- | --- | --- |
|  | Traditional UAS (n= 15) | S-UAS (n= 16) | p value | Traditional UAS (n= 38) | S-UAS (n= 34) | p value |
| SFR, n (%) | 6 (40%) | 13 (81.3%) | 0.018 | 26 (68.4%) | 28 (82.4%) | 0.173 |
| 1 month SFR, n (%) | 10 (66.7%) | 13 (81.3%) | 0.354 | 30 (78.9%) | 29 (87.9%) | 0.317 |
| Operative time (min) | 119 ± 49.94 | 135.31 ± 38.14 | 0.318 | 90.26 ± 34.09 | 77.89 ± 44.04 | 0.187 |
| Post-op hospital stays (hrs) | 2.67 ± 1.23 | 2.5 ± 1.32 | 0.719 | 2.84 ± 2.65 | 3.23 ± 4.37 | 0.653 |
| Post op serum Cr level (μmol/l) | 1.14 ± 0.48 | 1.13 ± 0.66 | 0.968 | 0.99 ± 0.29 | 1.01 ± 0.33 | 0.769 |
| Post op serum WBC count (103 /μl) | 12.17 ± 3.99 | 11.52 ± 3.92 | 0.652 | 10.53 ± 2.71 | 10.85 ± 4.87 | 0.732 |

**Supplement Table S2** Subgroup analysis of outcomes based on sum of stone size with cutoff value of 20mm

|  | Sum of stones > 20mm | | | Sum of stones <20mm | | |
| --- | --- | --- | --- | --- | --- | --- |
|  | Traditional UAS (n= 25) | S-UAS (n= 22) | p value | Traditional UAS (n= 28) | S-UAS (n= 28) | p value |
| SFR, n (%) | 14 (56%) | 15 (68.2%) | 0.391 | 18 (64.3%) | 26 (92.9%) | 0.009 |
| 1 month SFR, n (%) | 18 (72%) | 16 (76.2%) | 0.747 | 22 (78.6%) | 26 (92.9%) | 0.127 |
| Operative time (min) | 116.2 ± 41.16 | 121.36 ± 48.87 | 0.699 | 82.5 ± 33.95 | 76.59 ± 41.72 | 0.559 |
| Post-op hospital stays (hrs) | 3.28 ± 3.06 | 2.18 ± 0.91 | 0.098 | 2.36 ± 1.28 | 3.62 ± 4.77 | 0.178 |
| Post op serum Cr level (μmol/l) | 1.11 ± 0.42 | 1.14 ± 0.61 | 0.861 | 0.96 ± 0.28 | 0.98 ± 0.3 | 0.75 |
| Post op serum WBC count (103 /μl) | 11.38 ± 2.76 | 10.23 ± 3.79 | 0.246 | 10.65 ± 3.54 | 11.69 ± 5.05 | 0.373 |

**Supplement Table S3** Subgroup analysis of outcomes based on single or multiple stones

|  | **Single** | | | **Multiple** | | |
| --- | --- | --- | --- | --- | --- | --- |
|  | **Traditional UAS (n= 15)** | **S-UAS (n= 18)** | **p value** | **Traditional UAS (n= 38)** | **S-UAS (n= 32)** | **p value** |
| **SFR, n (%)** | 10 (66.7%) | 18 (100.0%) | 0.008 | 22 (57.9%) | 23 (71.9%) | 0.224 |
| **1 month SFR, n (%)** | 11 (73.3%) | 18 (100.0%) | 0.019 | 29 (76.3%) | 24 (77.4%) | 0.914 |
| **Operative time (min)** | 80.67 ± 37.31 | 76.84 ± 38.81 | 0.773 | 105.39 ± 40.51 | 107.22 ± 52.63 | 0.873 |
| **Post-op hospital stays (hrs)** | 2.93 ± 2.37 | 3.63 ± 5.66 | 0.631 | 2.74 ± 2.33 | 2.63 ± 1.72 | 0.818 |
| **Post op serum Cr level (μmol/l)** | 0.92 ± 0.29 | 0.99 ± 0.34 | 0.542 | 1.07 ± 0.38 | 1.08 ± 0.52 | 0.918 |
| **Post op serum WBC count (103 /μl)** | 10.63 ± 3.94 | 11.26 ± 5.68 | 0.708 | 11.15 ± 2.86 | 10.94 ± 3.85 | 0.802 |

**Supplement Table S4** Subgroup analysis of outcomes based on stone locations

|  | **Renal pelvis** | | | **upper/middle calyx** | | | **lower calyx** | | | **Upper ureter** | | | **Multiple or staghorn stone** | | |
| --- | --- | --- | --- | --- | --- | --- | --- | --- | --- | --- | --- | --- | --- | --- | --- |
|  | **Traditional UAS (n= 3)** | **S-UAS (n= 3)** | **p value** | **Traditional UAS (n= 9)** | **S-UAS (n= 6)** | **p value** | **Traditional UAS (n= 29)** | **S-UAS (n= 22)** | **p value** | **Traditional UAS (n= 3)** | **S-UAS (n= 7)** | **p value** | **Traditional UAS (n= 9)** | **S-UAS (n= 12)** | **p value** |
| **SFR, n (%)** | 2 (66.7%) | 3 (100%) | 0.273 | 7 (77.8%) | 5 (83.3%) | 0.792 | 16 (55.2%) | 18 (81.8%) | 0.046 | 2 (66.7%) | 7 (100%) | 0.107 | 5 (55.6%) | 8 (66.7%) | 0.604 |
| **1 month SFR, n (%)** | 2 (66.7%) | 3 (100%) | 0.273 | 8 (88.9%) | 5 (83.3%) | 0.756 | 21 (72.4%) | 18 (85.7%) | 0.262 | 2 (66.7%) | 7 (100%) | 0.107 | 7 (77.8%) | 9 (75%) | 0.882 |
| **Operative time (min)c** | 83.33 ± 20.82 | 66.67 ± 35.47 | 0.53 | 91.11 ± 38.22 | 58.33 ± 35.02 | 0.114 | 97.07 ± 38.28 | 99.36 ± 49.14 | 0.857 | 93.33 ± 75.72 | 80.63 ± 41.01 | 0.804 | 116.67 ± 46.7 | 125.83 ± 51.29 | 0.675 |
| **Post-op hospital stays (hrs)c** | 4.67 ± 4.62 | 3 ± 1.73 | 0.606 | 4.67 ± 4.09 | 2.17 ± 0.41 | 0.105 | 2.31 ± 1.23 | 3.05 ± 2.08 | 0.15 | 2.33 ± 1.15 | 5.13 ± 8.59 | 0.397 | 2 ± 0.71 | 1.92 ± 1 | 0.825 |
| **Post op serum Cr level (μmol/l)** | 1.05 ± 0.55 | 1.01 ± 0.23 | 0.915 | 0.95 ± 0.26 | 0.94 ± 0.11 | 0.929 | 1.01 ± 0.37 | 1.16 ± 0.62 | 0.339 | 1.17 ± 0.75 | 1.03 ± 0.44 | 0.836 | 1.13 ± 0.31 | 0.93 ± 0.21 | 0.109 |
| **Post op serum WBC count (103 /μl)** | 11.6 ± 0.46 | 14.13 ± 3.6 | 0.347 | 10.68 ± 2.98 | 10.53 ± 4.35 | 0.945 | 11.13 ± 3.42 | 9.92 ± 3.52 | 0.224 | 12 ± 5.53 | 12.23 ± 7.61 | 0.959 | 10.29 ± 2.54 | 11.87 ± 4.01 | 0.295 |
